# Structural Rearrangements of Polylactide/Natural Rubber Composites during Hydro- and Biotic Degradation

**DOI:** 10.3390/polym15081930

**Published:** 2023-04-19

**Authors:** Yulia V. Tertyshnaya, Maria V. Podzorova, Anastasia V. Khramkova, Vasily A. Ovchinnikov, Aleksey V. Krivandin

**Affiliations:** 1 Department of Biological and Chemical Physics of Polymers, Emanuel Institute of Biochemical Physics, Russian Academy of Sciences, 4 Kosygina Str., Moscow 119334, Russia; 2 Department of Chemistry of Innovative Materials and Technologies, Plekhanov Russian University of Economics, 36 Stremyanny per., Moscow 117997, Russia; 3 Politecnico di Milano, 32 Piazza Leonardo da Vinci, 20133 Milan, Italy

**Keywords:** polylactide, hydrolytic degradation, biodegradation, polymer composites, crystallinity, X-ray, Sturm test

## Abstract

In the work, the impact of the biological medium and water on structural rearrangements in pure polylactide and polylactide/natural rubber film composites was studied. Polylactide/natural rubber films with a rubber content of 5, 10, and 15 wt.% were obtained by the solution method. Biotic degradation was carried out according to the Sturm method at a temperature of 22 ± 2 °C. Hydrolytic degradation was studied at the same temperature in distilled water. The structural characteristics were controlled by thermophysical, optical, spectral, and diffraction methods. Optical microscopy revealed the surface erosion of all samples after exposure to microbiota and water. Differential scanning calorimetry showed a decrease in the degree of crystallinity of polylactide by 2–4% after the Sturm test, and a tendency to an increase in the degree of crystallinity after the action of water was noted. Changes in the chemical structure were shown in the spectra recorded by infrared spectroscopy. Due to degradation, significant changes in the intensities of the bands in the regions of 3500–2900 and 1700–1500 cm^−1^ were shown. The X-ray diffraction method established differences in diffraction patterns in very defective and less damaged regions of polylactide composites. It was determined that pure polylactide hydrolyzed more readily under the action of distilled water than polylactide/natural rubber composites. Film composites were more rapidly subjected to biotic degradation. The degree of biodegradation of polylactide/natural rubber composites increased with the rise in the content of natural rubber in the compositions.

## 1. Introduction

The degradation of polymers in aqueous media is a multifactorial process studied in physics, chemistry, and biology. Polylactide (PLA) and its degradation have attracted the attention of researchers for decades [1,2,3,4]. The use of new fillers and the creation of new polymer composites always left the question of biotic and abiotic degradation open.

Considering the process of biodegradation of polymers, it must be taken into account that this is not a monoprocess but a complex system of chemical and biological interactions, which may include hydrolysis and the action of enzymes secreted by microorganisms. There are also certain characteristics of the polymers themselves that affect the biodegradation process such as molecular weight and crystallinity. The high molecular weight and high crystallinity of PLA results in slower degradation [5,6].

The biodegradability and characteristics of the environment where the biodegradation process takes place are also of great importance. Unlike other biodegradable polymers, which degrade primarily in a single step by microbial attack, PLA degradation follows a sequential mechanism [7,8]. The first step involves reducing the molecular weight of PLA through hydrolysis, which is considered the rate-limiting step and is the main depolymerization mechanism. The second step involves microbial assimilation and metabolism of low molecular weight PLA oligomers and monomers [9]. The degree of degradation of aliphatic polyesters, to which PLA belongs, depends on the type and nature of enzymes produced by microorganisms [10]. The enzymes such as protease, esterase, and lipase produced by microbes show the ability to degrade PLA [11]. Proteinase and protease degrade polymer foils more rapidly than esterase and lipase [11,12].

However, studies [12,13,14,15] indicate that PLA-degrading bacteria are not as common in the environment. It was shown in the works [12,13] that PLA biodegradation mainly occurs due to microorganisms such as *Chryseobacterium* sp., *Sphingobacterium* sp., *Stenotrophomonas pavanii*, *Pseudomonas geniculata*, and *Thermopolyspora flexuosa*. In this case, the process inducers contain L-alanine units, which are similar to PLA L-lactic acid units in the stereochemical position of the chiral carbon [11]. Subsequently, the depolymerase acts on the intracellular ester bonds of PLA, resulting in the formation of oligomers, dimers and, consequently, monomers. Then, these low molecular weight compounds are absorbed by microorganisms, and they are converted into carbon dioxide, water, and methane as a result of the activity of intercellular enzymes [11,16,17,18]. However, as mentioned above, an important role for the biodegradation of PLA is played not only by the microorganisms themselves and hydrolytic enzymes but also by the chemical process of hydrolysis itself.

The course of the process of hydrolytic degradation occurs in several stages [19]:

- the diffusion of water into the material;
- the hydrolysis of chains in the amorphous region due to the lower resistance to water;
- the reducing of molecular weight due to the hydrolytic cleavage of side bonds and the formation of water-soluble compounds;
- the hydrolysis of the crystalline phase, which can occur by an autocatalytic mechanism due to acidic decomposition products as well as due to an increase in the concentration of carboxylic acid at the end of the chain.

The hydrolysis process is influenced by both external and internal factors. Important external factors are the temperature and pH of the medium.

It is known that an increase in temperature can accelerate the process of hydrolytic degradation. The temperature below which the PLA hydrolytic degradation rate decreases is 60 °C [19]. At a temperature of 37 °C, the rate of degradation is reduced by about two orders of magnitude compared with the process taking place at a temperature equal to or higher than 60 °C. This effect is associated with the transition of polylactide from a glassy to a highly elastic state, and the glass transition temperature of PLA is in the range of 58–63 °C.

In addition, such changes in the rates of hydrolytic degradation also occur in systems of mixtures of PLA with another component. For example, in a mixture of polylactide and polylactic-glycolic acid (in a ratio of 50:50), the rate of hydrolytic degradation under neutral conditions increases significantly at a process temperature above 60 °C [19].

The pH value is also important. It significantly affects the destruction of PLA since it affects not only the reaction mechanism but also the kinetics of the process. Destruction in an alkaline medium begins with a nucleophilic attack on the terminal hydroxyl of another carbonyl group. As a result of this process, oligomers of lactic acid and lactide are formed, which are degraded to lactic acid [20]. In an acidic environment, the destruction of polylactide is initiated by the protonation of the terminal ester group, after which an intramolecular hydrogen bridge is formed. The products of hydrolytic degradation by this mechanism are lactic acid and lactic acid oligomers [20]. As pH values increase from 1 to 10, the observed constant first decreases, reaching a minimum around pH 4, and then increases at higher pH values. At the same time, the growth of the constant is very significant—by about four orders of magnitude. The presence of a minimum in the dependence of the kinetic constant on pH confirms that hydrolysis is catalyzed either by an acidic or alkaline medium [19].

One of the most important internal factors is the crystallinity of the polymer [2,4]. The crystalline regions of PLA are resistant to hydrolysis due to the limited access of water molecules to the chains within the solid crystalline region. This causes selective hydrolytic chain cleavage in amorphous regions and the removal of water-soluble oligomers and monomers [21]. An increase in the concentration of D-units in PLA reduces optical purity and regularity, which leads to greater diffusion of water through the polymer matrix in amorphous regions and accelerated hydrolysis [21,22].

In a sample of complete amorphous PLA, a higher degree of destruction is observed in comparison with semicrystalline PLA under the same conditions [19,21]. A decrease in the molecular weight of amorphous PLA by 14% is observed after 18 weeks of being in a phosphate-buffered solution at pH = 4 and at a temperature of 37 °C. At the same time, it will take about 20 months to reduce the molecular weight of semi-crystalline PLA by the same value and under the same conditions [19].

Plasticizers and fillers also affect the process of PLA hydrolytic degradation. The reasons for the acceleration of the process may be the presence of a phase boundary, an increase in the proportion of the amorphous phase in the composite, and the hydrophilicity of the filler.

Rubbers are often used to increase the elasticity of brittle polymers [23,24,25]. Rubbers increase elongation at break and toughness of composites [24,26]. Natural rubber (NR) is an elastomer obtained from the sap of rubber trees. The application of NR makes it possible to improve some of the mechanical properties of PLA composites and preserve the bioavailability of the resulting material [27]. 

Most of the works are devoted to the study of morphological features and mechanical properties of PLA/NR. The structure changes of these composites during a degradation have not been sufficiently studied. It is expected that the natural rubber used in the current work will increase the rate of PLA hydro- and biotic degradation due to an increase in the proportion of the amorphous phase in the composites and the bioavailability of NR. The DSC method was used to determine the phase transition temperatures and the degree of crystallinity. Morphology was studied by optical microscopy. Changes in the chemical and physical structure were recorded by spectral methods and X-ray diffraction, respectively. 

## 2. Materials and Methods

### 2.1. Sample Preparation

Thermoplastic poly(lactic acid) (PLA) 4032D (with about 2% of D-lactide) with a molecular weight (M_w_) of 1.9 × 10^5^ g/mol was procured from Nature Works (Minnetonka, MN, USA). Natural rubber (NR), SVR-3L with a Mooney viscosity of 50 ± 5 (100 °C) and poly(cis-1,4-isoprene) content 91–96 by wt.% was kindly supplied by Vietnam Rubber Group (Ho Chi Minh City, Vietnam). The polylactide was previously dried at 40 °C for 3 h to remove moisture.

Film samples were obtained from a solution. The polymer solutions were prepared by dissolving PLA and PLA/NR in the right ratio in 100 mL of chloroform (Component-Reactiv, Moscow, Russia). The sample weight was 9 g per 100 mL, and the ratio of the components (PLA:NR, wt.%) was 100:0, 95:5, 90:10, and 85:15. The resulting samples were dried at 40 °C for 2.5 h to remove residual solvent.

### 2.2. Analysis of Crystallization

Thermal analysis was performed by differential scanning calorimeter (DSC) using a DSC 204 F1 device (Netzsch, Selb, Germany) under a nitrogen atmosphere. Samples of about 5.0–5.4 mg, sealed in aluminum pans, were heated from room temperature to 200 °C at a rate of 10 °C/min. Indium with T_m_ = 156.6 °C was used as a calibrant. The crystallinity of PLA (χ_c_) was estimated from the first heating cycle using the following equation:

χ_c_ (%) = 100% × (Δ*H*_m_/Δ*H*_m_*),

where Δ*H*_m_ is the enthalpy of melting during heating, and Δ*H*_m_* is the enthalpy assuming 100% crystalline PLA homopolymer 93.1 J/g [28].

### 2.3. FTIR Spectroscopy

The IR spectra were recorded on a Bruker Lumos IR Fourier device (Bruker Corp., Bremen, Germany) at a temperature of (21 ± 2) °C in the range of wave numbers 4000–400 cm^−1^. The analysis was carried out by attenuated total reflection (ATR) using a diamond crystal.

### 2.4. Morphology

The nonwoven fabrics’ morphologies were examined using an Olympus BX3M-PSLED (Tokyo, Japan) optical microscope of 200× in reflected light.

### 2.5. Biotic Degradation

The tests for the biodegradation of the samples under the action of soil microorganisms were carried out using a microbiological installation for the accelerated determination of biodegradation (modified Sturm method) (ISO 14855-1:2012).

The installation consisted of 18 parallel lines, twelve of which contained the analyzed samples, and the test temperature was 22 ± 2 °C. Each line consists of an air pump, six 500 mL Drexel bottles each, and a 1000 mL round bottom flask. Air was passed through the pump from left to right along each of the lines. In the first two Drexel flasks containing a concentrated (6 N) solution of sodium hydroxide, carbon dioxide was fixed from the air. The third and subsequent Drexel flasks contained a 0.05 N solution of Ba(OH)_2_. The tests were carried out for 90 days.

The air purified from carbon dioxide entered the reaction flask, which contained 500 mL of liquid soil inoculum with the test sample. The flask is closed from direct sunlight to avoid errors associated with the photosynthesis process. The soil inoculum preparation process used repeated filtration of the aqueous soil solution to get rid of the presence of protozoa in the solution.

The viability of bacteria, the presence of spores, and the absence of protozoa in the soil inoculum were analyzed using optical microscopy on a Micromed Polar 3 ToupCam 5.1 MP microscope (Micromed, St. Petersburg, Russia) at a magnification of 1000×.

During the operation of the biodegradation plant, carbon dioxide was continuously released by the microbiota in the reaction flask. The released carbon dioxide was captured in the fourth Drexel flask according to the reaction:

Ba(OH)_2_ + CO_2_ → BaCO_3_ + H_2_O

As a result of this reaction, the pH of the medium in the flask decreased from 8.5 to 8.0. To determine the amount of barium hydroxide reacted, a sample from the flask was titrated with 0.1 N hydrochloric acid to the equivalence point. The reaction of neutralization of barium hydroxide with hydrochloric acid is presented below:

Ba(OH)_2_ + 2HCl → BaCl_2_ + 2H_2_O

Based on the results of titration, carbon dioxide emission curves were constructed for each of the lines. Lines in which only the inoculum was present served as the baseline in the calculations. Lines in which starch was used as a biodegradable component served to confirm the viability of the microbiota. The percentage of biodegradation of a sample of polymeric material was calculated by the amount of released carbon relative to its content in the original sample.

### 2.6. Water Test

For testing, square-shaped film samples with a side of 25 mm were used. The test was carried out on three samples of each composition at temperature T = 22 ± 2 °C. The samples were placed in a vessel with distilled water, taken in an amount of at least 8 cm^3^ per 1 cm^2^ of the sample surface. The test samples did not come into contact with each other and were completely covered with distilled water. The test was carried out for 90 days.

### 2.7. X-ray Diffraction

An X-ray diffraction(Moscow, Russia) study of PLA based films was carried out in the Emanuel Institute of Biochemical Physics using an X-ray diffractometer of local design [29,30], supplied with the optical focusing of the X-ray beam and a linear position-sensitive X-ray detector. A fine-focus-sealed Cu X-ray tube with Ni β-filter was used as an X-ray source. The cross section of the X-ray beam in the plane of the sample was ~4 × 0.15 mm^2^. X-ray detector was installed with an inclination toward the sample at ~20°, the sample to detector distance was 92 mm, and the width of the detector window was restricted with a slit to be 4 mm in order to diminish the smearing of X-ray diffraction patterns. X-ray diffraction intensity was measured in the transmission mode in the range of the diffraction vector module 0.08 nm^–1^ < S < 6 nm^–1^, corrected for background scattering and normalized in such a way that the maximal intensity value observed at S ≈ 1.87 nm^–1^ had the value of 100 (S = 2sinθ/λ; 2θ: scattering angle; λ: X-ray wavelength; equal for CuKα radiation to 0.1542 nm).

### 2.8. Statistical Processing

The experimental results were calculated as the arithmetic mean and its standard error. The calculations were performed using Statistica 8.0 software (Dell Software Inc., Round Rock, TX, USA) and Microsoft Excel 2007.

## 3. Results and Discussion

The degradation of PLA by a generally accepted two-step mechanism first involved abiotic factors then biotic factors. The abiotic process, i.e., the chemical hydrolysis of PLA in the presence of water, after which biotic degradation occurs, in which microorganisms decompose polymer degradation products to form carbon dioxide, water, and biomass under aerobic conditions and methane, hydrocarbons, and biomass under anaerobic conditions [31,32,33].

**Figure 1:**
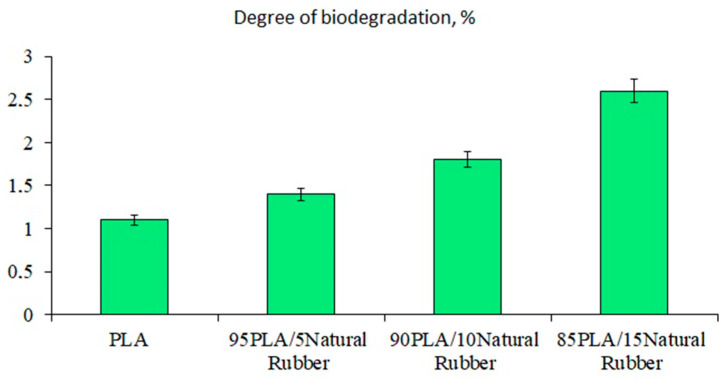
The degree of biodegradation of PLA/NR films at 22 ± 2 °C.

The biodegradation of PLA and PLA/NR composites was studied by the Sturm method. After 90 days of the experiment at room temperature, it was determined that the degree of biodegradation (Figure 1) was proportional to the content of NR in the composites.

The highest values were obtained for the composition containing 15 wt.% NR. This result was due to two reasons. First, NR is known to be degraded by enzymes. Microorganisms that destroy NR are widely distributed in the environment. Many bacterial strains have been studied that are able to use NR as the sole source of carbon and energy [34]. There are works on the study of the biochemical mechanism of the biological decomposition of natural rubber [35,36]. It was shown how easily raw NR became biodegradable under the influence of soil microorganisms. Thus, the weight loss of the NR film sample in wet laboratory soil for 90 days was 38 wt.% [37].

Secondly, PLA/NR composites are heterogeneous systems in which there is always an interface or an interfacial layer [38]. The interfacial layer and near-boundary regions are characterized by a reduced density compared to the component phases. Materials with such a structure are more actively attacked by aggressive media, thus composites can break down faster than pure polymers [27,39].

As for PLA, many studies have shown its slow degradation at room temperature. The process of the abiotic factors and biodegradation of PLA actively proceeds at a temperature above its glass transition temperature, since flexibility and water absorption increase, accelerating both hydrolysis and microbial attachment [19,40].

The structure and properties of the samples were controlled by DSC, X-ray, optical microscopy, and FTIR spectroscopy.

**Figure 2:**
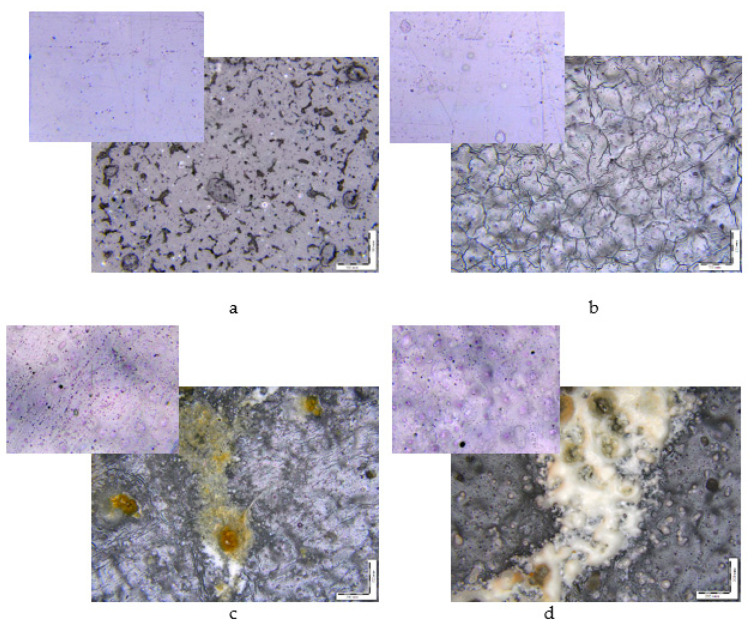
Micrographs of film samples with the NR content after the Sturm experiment, wt.%: (**a**) 0, (**b**) 5, (**c**) 10, (**d**) 15. The insets show the initial films of the same compositions. The magnification is 200×.

In Figure 2, images of initial samples (the inserts) and samples after biodegradation are shown. Changes in the morphologies of the composites are observed: crevices, cracks, and dark spots. The process of degradation of polymers always starts from the surface. When significant damage is formed in the structure of the surface layers, cracks penetrate the bulk of the material. At this stage, the properties of polymers deteriorate because the reactions of the decomposition of molecular chains proceed in the polymer matrix [24,41].

**Figure 3:**
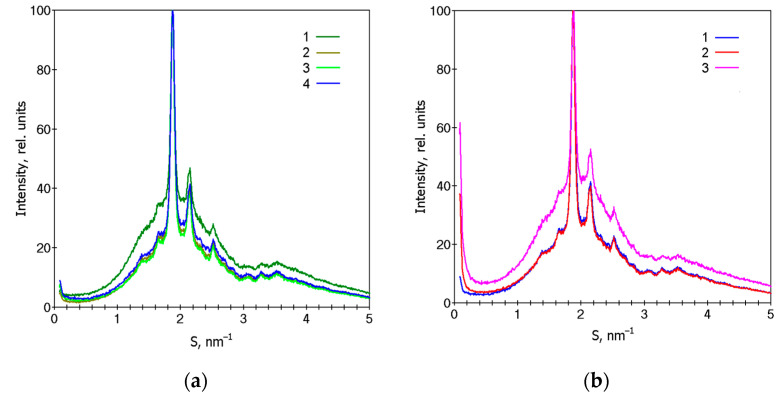
XRD patterns of film samples with the NR content, wt.%: (1) 0, (2) 5, (3) 10, (4) 15, (**a**) and the PLA85/NR15 film after the Sturm experiment: (1) initial, (2) less destroyed region, and (3) more destroyed region (**b**).

XRD tests were performed in all samples to study the structural changes after biodegradation. Figure 3a presents the XRD patterns of the initial samples. The degree of crystallinity of PLA/NR composites is higher than that of pure PLA (Figure 3a, curve 1).

It can be seen that all films have a diffraction peak at S = 1.87 nm^−1^ and two weak peaks at S = 2.14 and 2.78 nm^−1^, which are related to the α-form of crystallized PLA [42,43]. Then, XRD analysis was carried out in the damaged spot of the film composites and the less destroyed region (Figure 3b). Figure 3b shows the patterns of the 85PLA/15NR sample, and a similar relation is observed for all composites. As a result of processing the samples by the Sturm method, for their main part, which does not contain visible macroscopic defects, the degree of crystallinity and the crystalline form of PLA change little, but, judging by the rising in the intensity of small-angle X-ray scattering at S < 0.2 nm^–1^, the nanoscale inhomogeneity of their structure increases. The diffraction pattern obtained from the region with a macroscopic film defect (Figure 3b, curve 3) indicates not only an increase in the nanoscale inhomogeneity of its structure but also a significant decrease in the degree of crystallinity in this region of the film compared to the main part without such defects.

**Figure 4:**
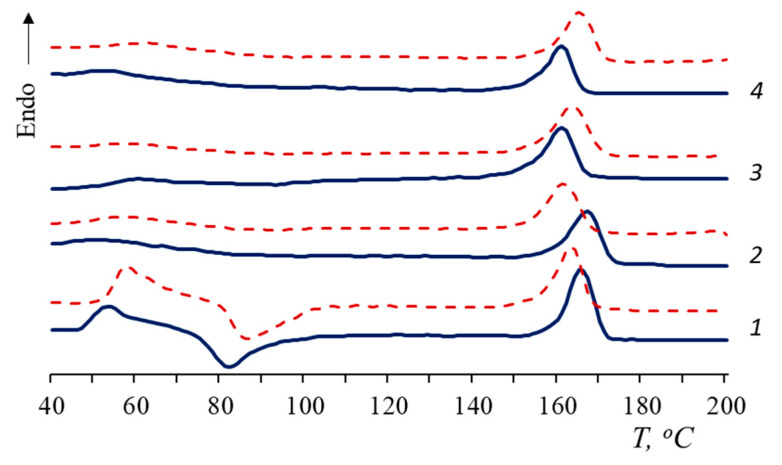
Melting thermograms of PLA/NR films. The content of NR, wt.%: (1) 0; (2) 5; (3) 10; and (4) 15. Blue: initial samples; red: after biotic degradation.

In order to gain information about thermophysical properties, the film composites were also investigated using DSC. In Figure 4, the thermograms of the samples are represented. In total, 100% PLA has a cold crystallization peak, thus the degree of crystallinity, calculated as the difference between the enthalpies of melting and cold crystallization, is less than the χ value of PLA composites (Table 1). The DSC results are in agreement with the X-ray data.

According to the DSC curves, when NR is added to the PLA matrix, the cold crystallization peak disappears. This is associated with an increase in the total amount of the amorphous phase in the composites and, probably, with an increase in the segmental mobility of macromolecules [27]. In the presence of NR, PLA molecular chains have time to fit into domains and form a crystalline structure that does not require recrystallization. 

The shift of T_cc_ towards a lower temperature could be attributed to a decrease in the molecular weight and rupture of the PLA macrochains [44]. However, in the current work, an inverse relationship was observed: in the process of degradation, the T_cc_ of PLA increased.

**Figure 5:**
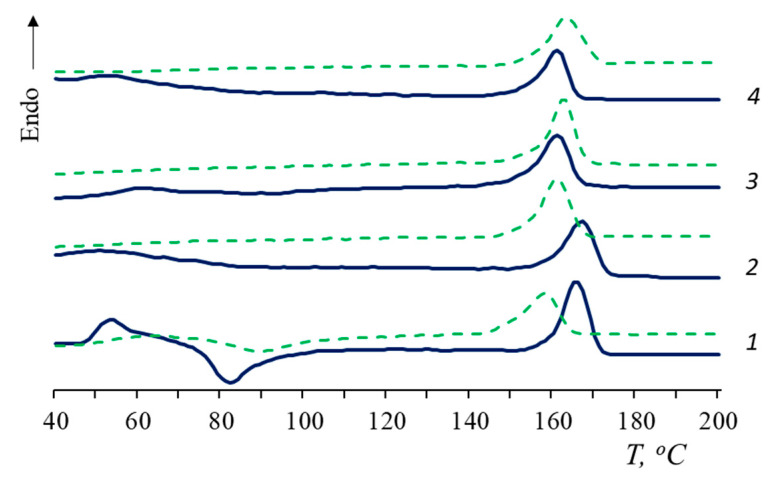
Melting thermograms of PLA/NR films. The content of NR, wt.%: (1) 0; (2) 5; (3) 10;.(4) 15. Blue: initial samples; green: after hydrolytic degradation.

The glass transition temperature (T_g_) of PLA/NR composites becomes difficult to determine (values are given in brackets). The T_g_ values of PLA/NR composites are 2–3 °C higher than 100% PLA ones. After degradation for 90 days, the glass transition temperature of PLA disappears in all PLA/NR composites (Figure 4 and Figure 5). This behavior could be explained by the effect of plasticization [45]. Luo et al. observed the disappearance of the cold crystallization peak of PLA composites after 135 days of hydrolytic degradation [45].

After biotic and hydrolytic degradation, the melting point of PLA decreases. Water has a greater effect on PLA T_m_ than the microbiota. It would seem that the polymer should break down faster in the process of biodegradation. However, it is known that the activity of the microbiota increases at temperatures of 30 °C or more [46,47], while PLA undergoes hydrolytic cleavage at an ambient temperature, and the process accelerates by increasing the temperature.

According to Table 1, the trend in the change in the degree of crystallinity is also different. After the Sturm test, the PLA degree of crystallinity decreases in all samples by 2–4%. After the action of distilled water, there is a tendency to increase the degree of PLA crystallinity. These are interesting results of the degradation of heterogeneous systems that require further investigation at various temperatures.

**Figure 6:**
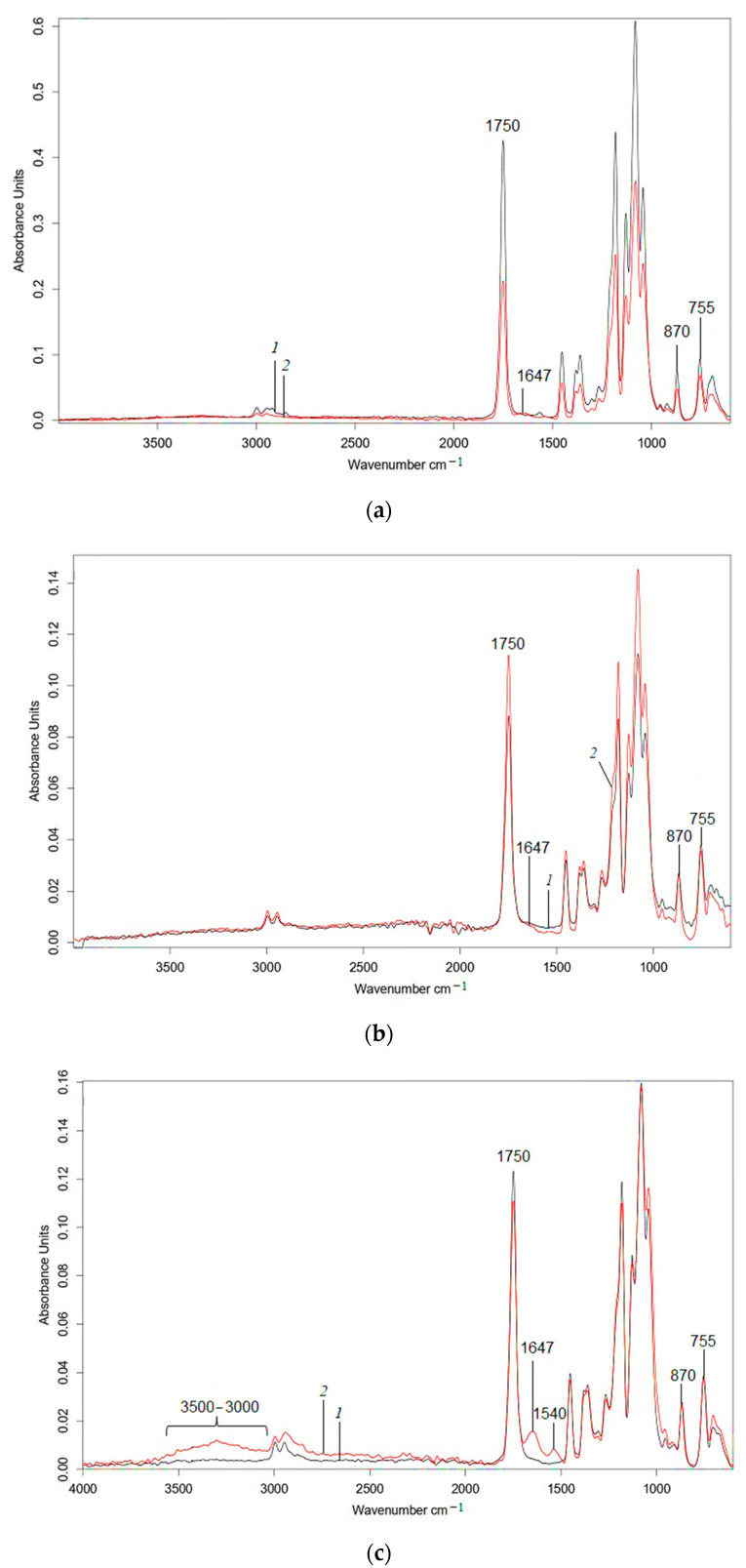

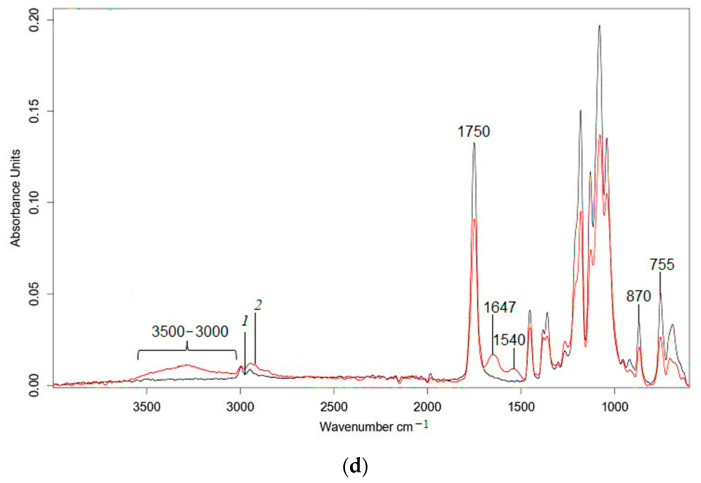
FTIR-ATR spectra of PLA/NR films. The content of NR, wt.%: (**a**) 0; (**b**) 5; (**c**) 10; (**d**) 15. Black: initial samples (1); red: after biotic degradation (2).

Changes in the chemical structure of PLA and PLA composites were studied by FTIR-ATR spectroscopy. Analysis of IR spectra of PLA/NR composites (Figure 6) showed a high absorption intensity in the region of 1740–1080 cm^−1^, which corresponded to different vibrations of polylactide fragments [48,49,50]. As a result of exposure to soil bacteria, the intensity of structure-sensitive bands decreased for almost all samples. In the region of 1380–1000 cm^−1^, where the active C–O– groups were located, the absorption intensity decreased due to the disintegration of ester groups as a result of the degradation process. In the 1450 cm^−1^ region, related to asymmetric vibrations of the methyl group CH_3_– referring to PLA, a change in intensity was also observed [48].

It is worth mentioning that the greatest change in the absorption intensity occurred for the sharp peak in the 1750 cm^−1^ region, which belongs to the valent vibrations of the C=O carbonyl group in the aliphatic ethers. This fact clearly indicates the disintegration of the polylactide phase in all samples under the action of the soil environment [49]. 

The intensity of the structure-sensitive band 755 cm^−1^, related to the –C–C– oscillations of the polylactide crystalline phase, decreased, confirming its destruction in the process of microorganism acting [50]. The reduction in the intensity of the 755 cm^−1^ band correlated with the DSC results. After the Sturm experiment, the PLA degree of crystallinity decreased (Table 1). It should be mentioned that the 870 cm^−1^ band, corresponding to –C–C– vibrations of the polylactide amorphous phase, overlapped with the 836 cm^−1^ peak, related to the C–H vibrations of the (CH_3_)C=CH– rubber molecule group. The decrease in absorption intensity in this region proved the degradation of both the PLA and NR.

With the increase in the NR content in the composites, the appearance of two peaks in the interval 1660–1500 cm^−1^ was observed. This is the result of microorganism interaction with a natural rubber molecule leading to C–N and N–H amide groups formation, while their vibrations increased the absorption intensity in this region [51]. The authors of the study on the biodegradation process of polyethylene/natural rubber (PE/NR) blends [37] also observed the appearance of bands in this region. In PE/NR samples, as well as in the studied samples, the intensity of bands in the region of 1660–1500 cm^−1^ increased with the rising NR content.

In addition, a broad band appeared in the region of 3500–3000 cm^−1^ corresponding to bound and single vibrations of the –OH groups. They were formed as a result of the chain oxidation under the influence of oxogenases on the polymer sample. As the concentration of natural rubber in the composites increased, the intensity of vibrations in this region enhanced significantly, since the rubber molecule underwent easier splitting due to its amorphous structure [52]. The disintegration of NR could be traced in the proton NMR spectra (Figure S1).

**Figure 7:**
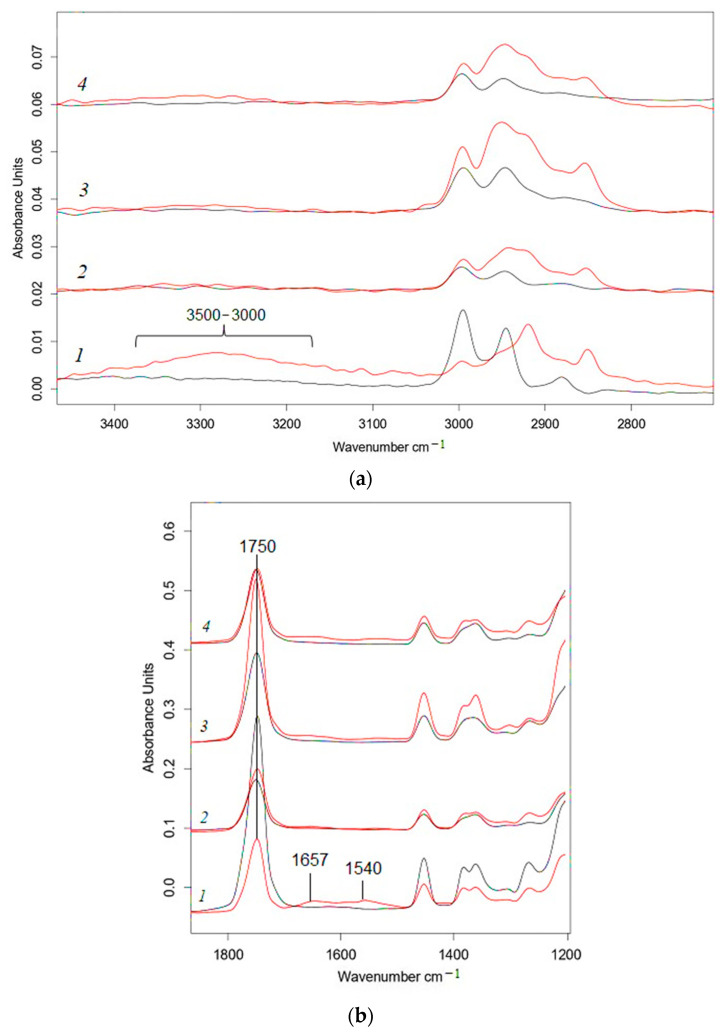
FTIR-ATR spectra of PLA/NR films: the region 3500–2800 cm^−1^ (**a**) and the region 1800–1200 cm^−1^ (**b**). The content of NR, wt.%: (1) 0; (2) 5; (3) 10; (4) 15. Black—initial samples, red—after hydrolytic degradation.

As mentioned above, distilled water and microbiota affected the process of the disintegration of PLA/NR composites in different ways. In contrast to the Sturm test in the region of 3500–3100 cm^−1^, a slight increase in the intensity of the band was observed only for PLA (Figure 7a), which referred to the formation of –OH groups during hydrolysis. After exposure to water, peaks at 1657 and 1540 cm^−1^ were not traced for PLA/NR composites, and there was a slight increase in intensity in this region only for 100% PLA (Figure 7b).

In the spectrum of pure PLA, the intensity of the band at 1750 cm^−1^ decreases (Supplementary File). Adding natural rubber changes the dependency. In the spectra of all PLA/NR composites, the intensity of the band at 1750 cm^−1^ increased. This meant that the formation of C=O groups of acids and esters during degradation prevailed over the process of elimination of PLA ester groups when NR added.

**Figure 8:**
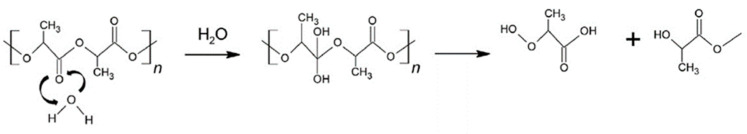
Mechanism of PLA hydrolysis in distilled water.

The morphologies of the samples also changed. The PLA/NR film composites became muddy. This result was not surprising. Many authors observed a similar effect [2,3,6,10,45]. It is known that oligomers, dimers, and lactic acid are formed (Figure 8) in the process of PLA hydrolytic degradation, which cause a loss of transparency.

Summarizing the results, the influence of microbiota and water, even at ambient temperature, on PLA and PLA/NR composites becomes evident. Despite a low degree of biodegradation, the morphologies and structures of the samples changed, which was recorded by various methods. In the middle of the 20th century, in the works of J.A. Manson, L.H. Sperling, V.N. Kuleznev, and other authors, the features of the structures and the properties of heterogeneous polymer systems were studied. Definitely, these scientists did not work with biodegradable polymers, but, nevertheless, the patterns remained the same. Stresses were formed at the polymer1-polymer2 interface due to different viscosities, thermal expansion coefficients of the components, the chemical nature of the polymers, and other parameters. As a result, very often polymer blends and composites degraded faster than homopolymers.

## 4. Conclusions

PLA and PLA/NR film composites with 5, 10, and 15 wt.% NR were obtained and subjected to biotic and hydrolytic degradation at an ambient temperature. From the mentioned results, the following conclusions can be derived:

- As it stands, 100% PLA is well hydrolyzed in distilled water, and PLA/NR films are more actively subjected to biotic degradation compared to pure polylactide;
- The influence of natural rubber on the structure and thermophysical characteristics of the PLA matrix and on the behavior of PLA/NR composites during degradation is shown;
- The morphological observation indicated the surface erosion of all samples both during biotic and hydrolytic degradation;
- Differences in the crystal structure of PLA in highly defective and less damaged film regions were detected by the XRD method;
- FTIR-ATP spectra demonstrated a significant change in the chemical structure of PLA/NR film composites during hydro- and bio-degradation;
- Adding NR increases the degree of biodegradation of PLA/NR composites due to the formation of a heterogeneous system and an increase in the proportion of the amorphous phase in the samples.

## Figures and Tables

**Table 1:** Thermophysical characteristics of PLA/NR film samples: before and after biotic and hydrolytic degradation.

NR Content, wt.%	T_g_, °C (Δ ± 0.5 °C)	T_g_, °C (Δ ± 0.5 °C) Sturm	T_g_, °C (Δ ± 0.5 °C) Water	T_cc_, °C (Δ ± 0.5 °C)	T_cc_, °C (Δ ± 0.5 °C) Sturm	T_cc_, °C (Δ ± 0.5 °C) Water	T_m_, °C (Δ ± 0.5 °C)	T_m_, °C (Δ ± 0.5 °C) Sturm	T_m_, °C (Δ ± 0.5 °C) Water	χ, % (Δ ± 0.5%)	χ, % (Δ ± 0.5%) Sturm	χ, % (Δ ± 0.5%) Water
0	48	52	-	81	85	89	165	163	158	22	20	23
5	(48)	-	-	-	-	-	165	162	161	31	29	31
10	(50)	-	-	-	-	-	161	163	163	30	28	32
15	(51)	-	-	-	-	-	161	164	164	30	26	30

## Data Availability

The data presented in this study are available on request from the corresponding author.

## Supplementary Materials

The following supporting information can be downloaded at: https://www.mdpi.com/article/10.3390/polym15081930/s1, Figure S1. FTIR-ATR spectra of PLA/NR films. The content of NR, wt.%: (a) 0; (b) 5; (c) 10; (d) 15. Black—initial samples; Figure S2. ^1^H NMR spectra of PLA/NR films. The content of NR, wt.%: (a,b) 0; (c,d) 5; (e,f) 10; (g,h) 15.

## References

[1] Piemonte V., Gironi F. (2013). Kinetics of Hydrolytic Degradation of PLA. J. Polym. Environ..

[2] Tertyshnaya Y.V., Karpova S.G., Popov A.A. (2017). Effect of Aqueous Medium on the Molecular Mobility of Polylactide. Russ. J. Phys. Chem. B.

[3] Deroiné M., le Duigou A., Corre Y.M., le Gac P.Y., Davies P., César G., Bruzaud S. (2014). Accelerated Ageing of Polylactide in Aqueous Environments: Comparative Study between Distilled Water and Seawater. Polym. Degrad. Stab..

[4] Tertyshnaya Y., Podzorova M., Moskovskiy M. (2021). Impact of Water and UV Irradiation on Nonwoven Polylactide/Natural Rubber Fiber. Polymers.

[5] Feng P., Jia J., Liu M., Peng S., Zhao Z., Shuai C. (2021). Degradation Mechanisms and Acceleration Strategies of Poly (Lactic Acid) Scaffold for Bone Regeneration. Mater. Des..

[6] Rosli N.A., Karamanlioglu M., Kargarzadeh H., Ahmad I. (2021). Comprehensive Exploration of Natural Degradation of Poly(Lactic Acid) Blends in Various Degradation Media: A Review. Int. J. Biol. Macromol..

[7] Karamanlioglu M., Preziosi R., Robson G.D. (2017). Abiotic and Biotic Environmental Degradation of the Bioplastic Polymer Poly(Lactic Acid): A Review. Polym. Degrad. Stab..

[8] Kliem S., Kreutzbruck M., Bonten C. (2020). Review on the Biological Degradation of Polymers in Various Environments. Materials.

[9] Iozzino V., Askanian H., Leroux F., Verney V., Pantani R. (2018). Poly(Lactic Acid)-Based Nanobiocomposites with Modu-lated Degradation Rates. Materials.

[10] Karamanlioglu M., Robson G.D. (2013). The Influence of Biotic and Abiotic Factors on the Rate of Degradation of Poly(Lactic) Acid (PLA) Coupons Buried in Compost and Soil. Polym. Degrad. Stab..

[11] Richert A., Dąbrowska G.B. (2021). Enzymatic Degradation and Biofilm Formation during Biodegradation of Polylactide and Polycaprolactone Polymers in Various Environments. Int. J Biol. Macromol..

[12] Bubpachat T., Sombatsompop N., Prapagdee B. (2018). Isolation and role of polylactic acid-degrading bacteria on degrading enzymes productions and PLA biodegradability at mesophilic conditions. Polym. Degrad. Stab..

[13] Decorosi F., Exana M.L., Pini F., Adessi A., Messini A., Giovannetti L., Viti C. (2019). The Degradative Capabilities of New *Amycolatopsis* Isolates on Polylactic Acid. Microorganisms.

[14] Muroi F., Tachibana Y., Soulenthone P., Yamamoto K., Mizuno T., Sakurai T., Kobayashi Y., Kasuya K.i. (2017). Characterization of a Poly(Butylene Adipate-Co-Terephthalate) Hydrolase from the Aerobic Mesophilic Bacterium *Bacillus Pumilus*. Polym. Degrad. Stab..

[15] Odobel C., Dussud C., Philip L., Derippe G., Lauters M., Eyheraguibel B., Burgaud G., ter Halle A., Meistertzheim A.L., Bruzaud S. (2021). Bacterial Abundance, Diversity and Activity During Long-Term Colonization of Non-Biodegradable and Biodegradable Plastics in Seawater. Front. Microbiol..

[16] Gu J.D. (2003). Microbiological Deterioration and Degradation of Synthetic Polymeric Materials: Recent Research Advances. Int. Biodeterior. Biodegrad..

[17] Tokiwa Y., Calabia B.P. (2006). Biodegradability and Biodegradation of Poly(Lactide). Appl. Microbiol. Biotech..

[18] Tarach I., Olewnik-Kruszkowska E., Richert A., Gierszewska M., Rudawska A. (2020). Influence of Tea Tree Essential Oil and Poly(Ethylene Glycol) on Antibacterial and Physicochemical Properties of Polylactide-Based Films. Materials.

[19] Gorrasi G., Pantani R., Di Lorenzo M.L., Androsch R. (2018). Hydrolysis and Biodegradation of Poly(lactic acid). Synthesis, Structure and Properties of Poly(lactic acid).

[20] Olewnik-Kruszkowska E. (2016). Influence of the Type of Buffer Solution on Thermal and Structural Properties of Polylactide-Based Composites. Polym. Degrad. Stab..

[21] Lim B.K.H., Thian E.S. (2022). Biodegradation of Polymers in Managing Plastic Waste—A Review. Sci. Total Environ..

[22] Li S., Girard A., Garreau H., Vert M. (2000). Enzymatic Degradation of Polylactide Stereocopolymers with Predominant D-Lactyl Contents. Polym. Degrad. Stab..

[23] Si W.J., Yuan W.Q., Li Y.D., Chen Y.K., Zeng J.B. (2018). Tailoring Toughness of Fully Biobased Poly(Lactic Acid)/Natural Rubber Blends through Dynamic Vulcanization. Polym. Test..

[24] Tertyshnaya Y.V., Podzorova M.V., Varyan I.A., Tcherdyntsev V.V., Zadorozhnyy M.Y., Medvedeva E.V. (2023). Promising Agromaterials Based on Biodegradable Polymers: Polylactide and Poly-3-Hydroxybutyrate. Polymers.

[25] Pongtanayut K., Thongpin C., Santawitee O. (2013). The Effect of Rubber on Morphology, Thermal Properties and Mechanical Properties of PLA/NR and PLA/ENR Blends. Energy Procedia.

[26] Kowalczyk M., Piorkowska E. (2012). Mechanisms of Plastic Deformation in Biodegradable Polylactide/Poly(1,4- Cis-Isoprene) Blends. J. Appl. Polym. Sci..

[27] Tertyshnaya Y.V., Karpova S.G., Podzorova M.V., Khvatov A.V., Moskovskiy M.N. (2022). Thermal Properties and Dynamic Characteristics of Electrospun Polylactide/Natural Rubber Fibers during Disintegration in Soil. Polymers.

[28] Auras R., Harte B., Selke S. (2004). An Overview of Polylactides as Packaging Materials. Macromol. Biosci..

[29] Krivandin A.V., Solov’Eva A.B., Glagolev N.N., Shatalova O.V., Kotova S.L. (2003). Structure Alterations of Perfluorinated Sulfocationic Membranes under the Action of Ethylene Glycol (SAXS and WAXS Studies). Polymer.

[30] Krivandin A.V., Fatkullina L.D., Shatalova O.V., Goloshchapov A.N., Burlakova E.B. (2013). Small-Angle X-Ray Scattering Study of the Incorporation of ICHPHAN Antioxidant in Liposomes. Russ. J. Phys. Chem. B.

[31] Ghorpade V.M., Gennadios A., Hanna M.A. (2001). Laboratory Composting of Extruded Poly(Lactic Acid) Sheets. Bioresour. Technol..

[32] Longieras A., Tanchette J.B., Erre D., Braud C., Copinet A. (2007). Compostability of Poly(Lactide): Degradation in an Inert Solid Medium. J. Polym. Environ..

[33] Itävaara M., Karjomaa S., Selin J.F. (2002). Biodegradation of Polylactide in Aerobic and Anaerobic Thermophilic Conditions. Chemosphere.

[34] Yikmis M., Steinbüchel A. (2012). Historical and Recent Achievements in the Field of Microbial Degradation of Natural and Synthetic Rubber. Appl. Environ. Microbiol..

[35] Ali Shah A., Hasan F., Shah Z., Kanwal N., Zeb S. (2013). Biodegradation of Natural and Synthetic Rubbers: A Review. Int. Biodeterior. Biodegrad..

[36] Nanthini J., Sudesh K. (2017). Biodegradation of Natural Rubber and Natural Rubber Products by *Streptomyces* Sp. Strain CFMR 7. J. Polym. Environ..

[37] Varyan I., Kolesnikova N., Xu H., Tyubaeva P., Popov A. (2022). Biodegradability of Polyolefin-Based Compositions: Effect of Natural Rubber. Polymers.

[38] Karpova S.G., Tertyshnaya Y.V., Podzorova M.V., Popov A.A. (2021). Effect of Exposure in Aqueous Medium at Elevated Temperature on the Structure of Nonwoven Materials Based on Polylactide and Natural Rubber. Polym. Sci. Ser. A.

[39] Kuleznev V.N., Shershnev V.A. (2014). Chemistry and Physics of Polymers.

[40] Satti S.M., Shah A.A., Auras R., Marsh T.L. (2017). Isolation and Characterization of Bacteria Capable of Degrading Poly(Lactic Acid) at Ambient Temperature. Polym. Degrad. Stab..

[41] Kale G., Auras R., Singh S.P. (2007). Comparison of the Degradability of Poly(Lactide) Packages in Composting and Ambient Exposure Conditions. Pack. Technol. Sci..

[42] Podzorova M.V., Tertyshnaya Y.V., Ziborov D.M., Poletto M. (2020). Damage of Polymer Blends Polylactide-Polyethylene under the Effect of Ultraviolet Irradiation. AIP Conf. Proc..

[43] Hoogsteen W., Postema A.R., Pennings A.J., ten Brinke G., Zugenmaier P. (1990). Crystal Structure, Conformation, and Morphology of Solution-Spun Poly(L-Lactide) Fibers. Macromolecules.

[44] Yasuniwa M., Tsubakihara S., Iura K., Ono Y., Dan Y., Takahashi K. (2006). Crystallization Behavior of Poly(l-Lactic Acid). Polymer.

[45] Luo Y.B., Wang X.L., Wang Y.Z. (2012). Effect of TiO_2_ Nanoparticles on the Long-Term Hydrolytic Degradation Behavior of PLA. Polym. Degrad. Stab..

[46] Noor H., Satti S.M., ud Din S., Farman M., Hasan F., Khan S., Badshah M., Shah A.A. (2020). Insight on Esterase from *Pseudomonas Aeruginosa* Strain S3 That Depolymerize Poly(Lactic Acid) (PLA) at Ambient Temperature. Polym. Degrad. Stab..

[47] Mistry A.N., Kachenchart B., Wongthanaroj A., Somwangthanaroj A., Luepromchai E. (2022). Rapid Biodegradation of High Molecular Weight Semi-Crystalline Polylactic Acid at Ambient Temperature via Enzymatic and Alkaline Hydrolysis by a Defined Bacterial Consortium. Polym. Degrad. Stab..

[48] Ndazi B.S., Karlsson S. (2011). Characterization of Hydrolytic Degradation of Polylactic Acid/Rice Hulls Composites in Water at Different Temperatures. Express Polym. Lett..

[49] Arrieta M.P., López J., Rayón E., Jiménez A. (2014). Disintegrability under Composting Conditions of Plasticized PLA–PHB Blends. Polym. Degrad. Stab..

[50] Copinet A., Bertrand C., Govindin S., Coma V., Couturier Y. (2004). Effects of Ultraviolet Light (315 Nm), Temperature and Relative Humidity on the Degradation of Polylactic Acid Plastic Films. Chemosphere.

[51] Maquelin K., Kirschner C., Choo-Smith L.-P., van den Braak N., Endtz H.P., Naumann D., Puppels G.J. (2002). Identification of Medically Relevant Microorganisms by Vibrational Spectroscopy. J. Microbiol. Methods.

[52] Schmitt J., Flemming H.C. (1998). FTIR-Spectroscopy in Microbial and Material Analysis. Int. Biodeterior. Biodegrad..

