# Supplementary material for: Structural Rearrangements of Polylactide/Natural Rubber Composites during Hydro- and Biotic Degradation
Source: Polymers (Basel). 2023 Apr 19;15(8):1930. doi: 10.3390/polym15081930 (PMC10145913; doi:10.3390/polym15081930)
Supplement: Supplementary file 1 [file polymers-15-01930-s001.zip › polymers-2279635-supplementary.pdf]

SUPPLEMENTARY FILE (Tertyshnaya Yulia *et al.*)

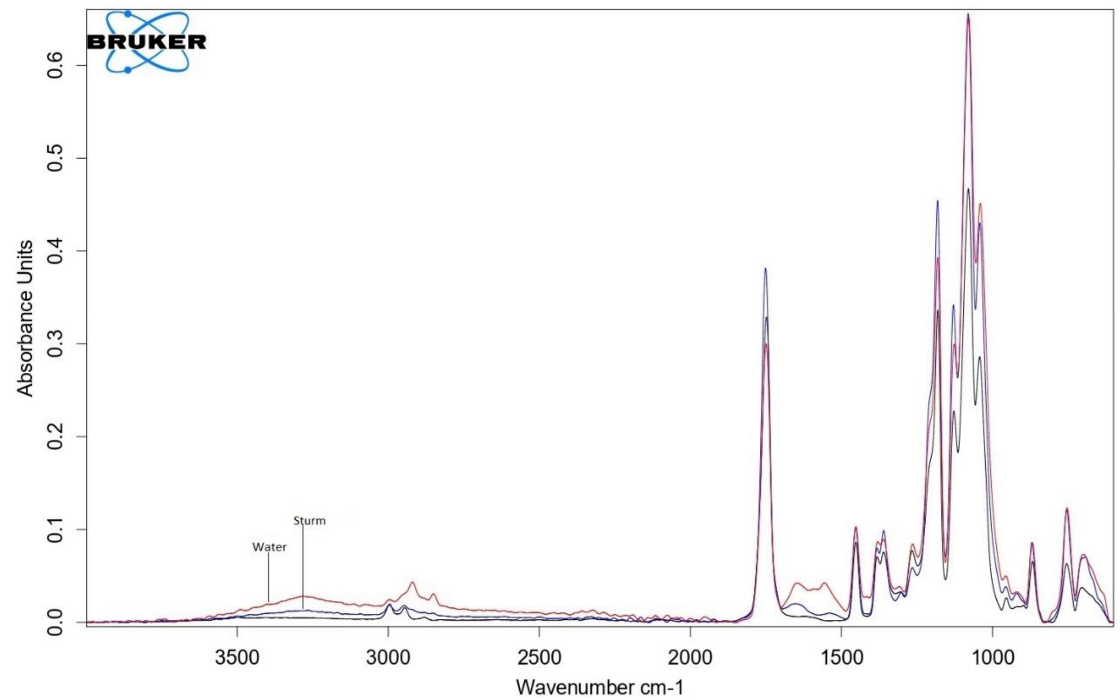

100% PLA (a)

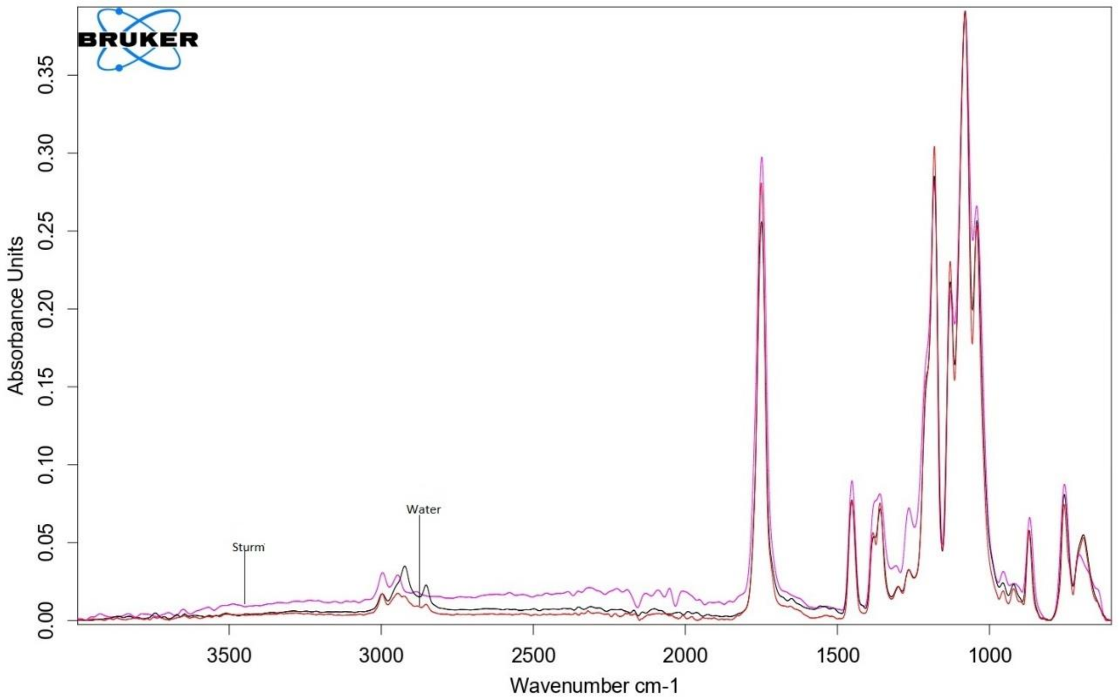

95PLA/5NR (b)

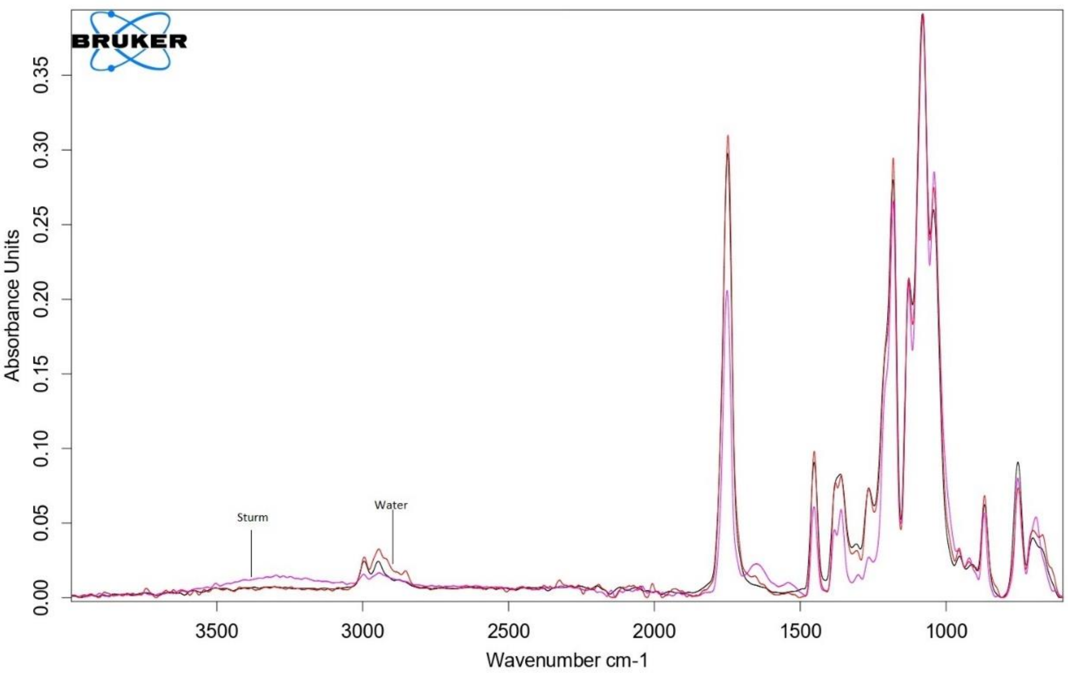

90PLA/10NR (c)

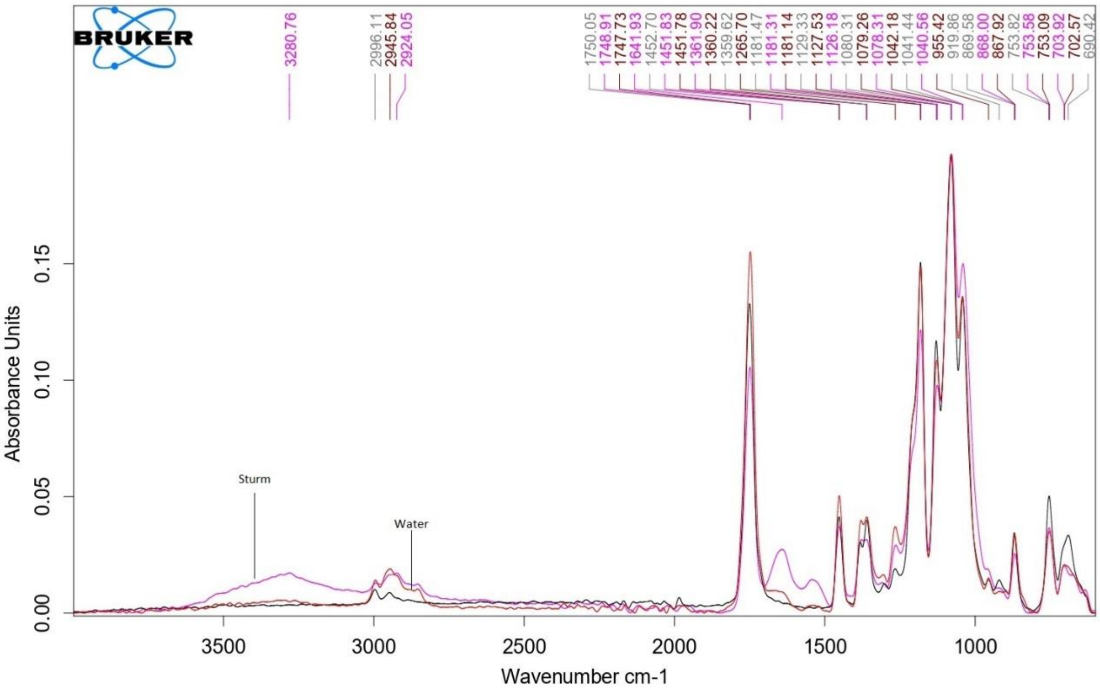

85PLA/15NR (d)

Figure S1. FTIR-ATR spectra of PLA/NR films. The content of NR, wt.%: a) 0; b) 5; c) 10; d) 15. Black – initial samples.

^{1}H NMR spectra

In the ^{1}H NMR spectra, the two typical peaks at 1.60 ppm and 5.13-5.24 ppm are associated with the hydrogens of CH_3 and CH from PLA respectively. The peak at 7.2 ppm is attributed to the solvent (deuterated chloroform).

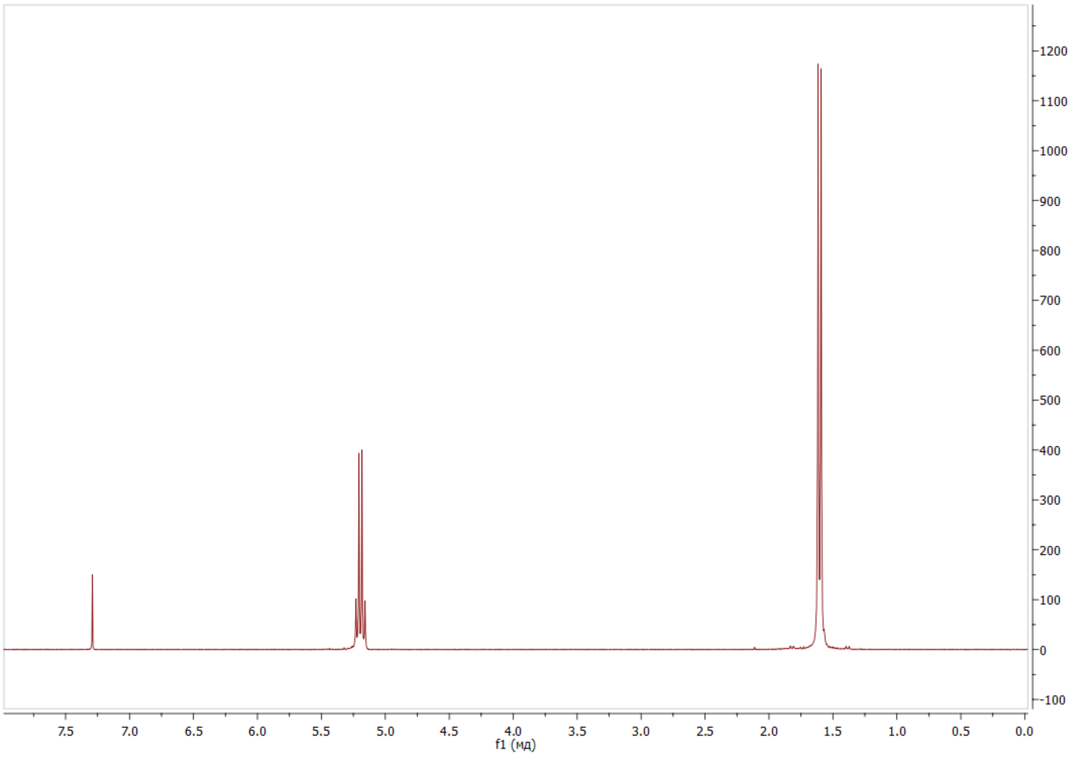

100% PLA, *initial* (a)

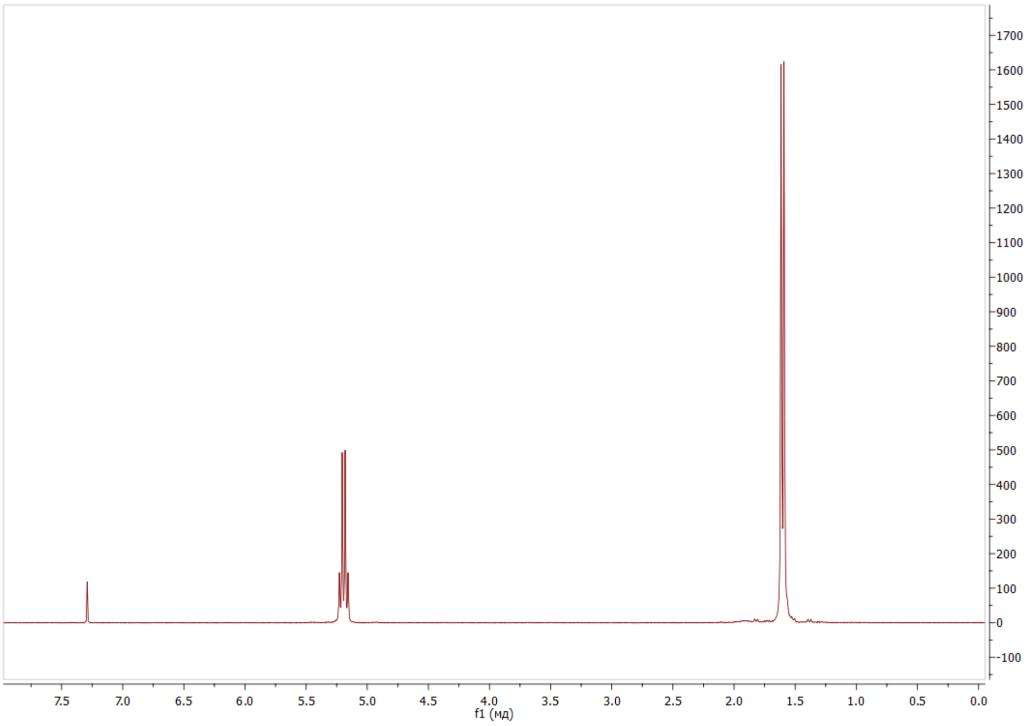

100% PLA, *Sturm method* (b)

Two additional signals 1.7 ppm (hydrogens of methyl groups) and 2.1 ppm (hydrogens of methylene groups) of natural rubber appear on the PLA/NR spectra. The peak of the unsaturated methylene protons of the cis-units of 1,4-isoprene overlaps with the PLA protons.

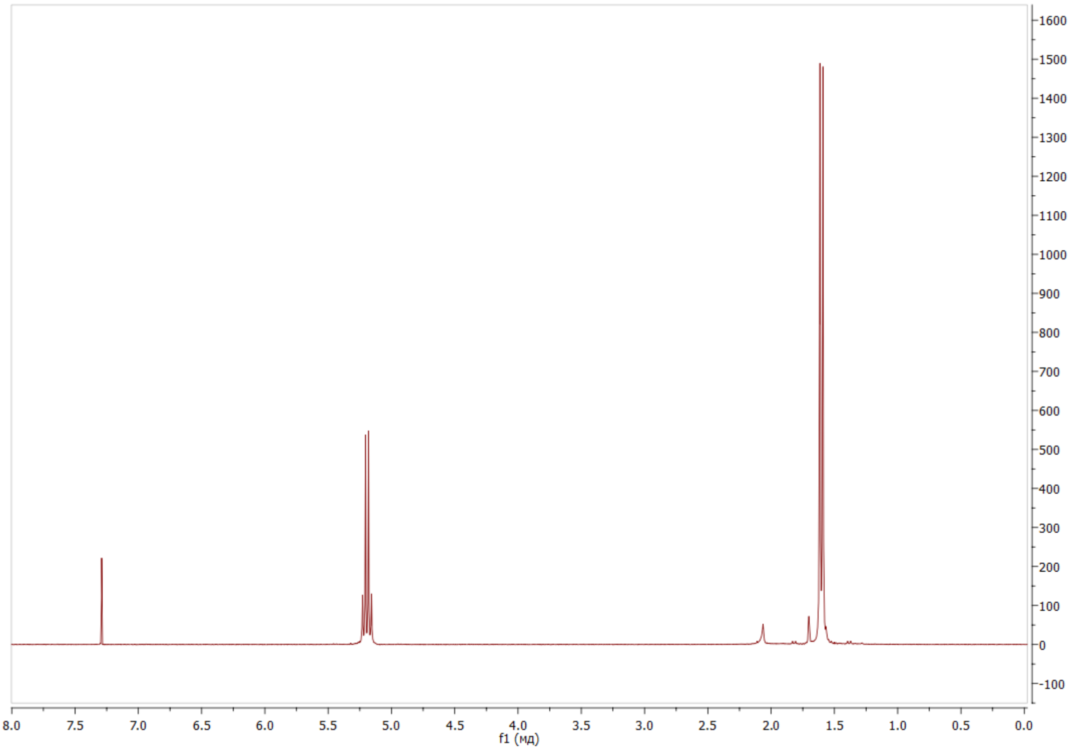

95PLA/5NR, *initial* (c)

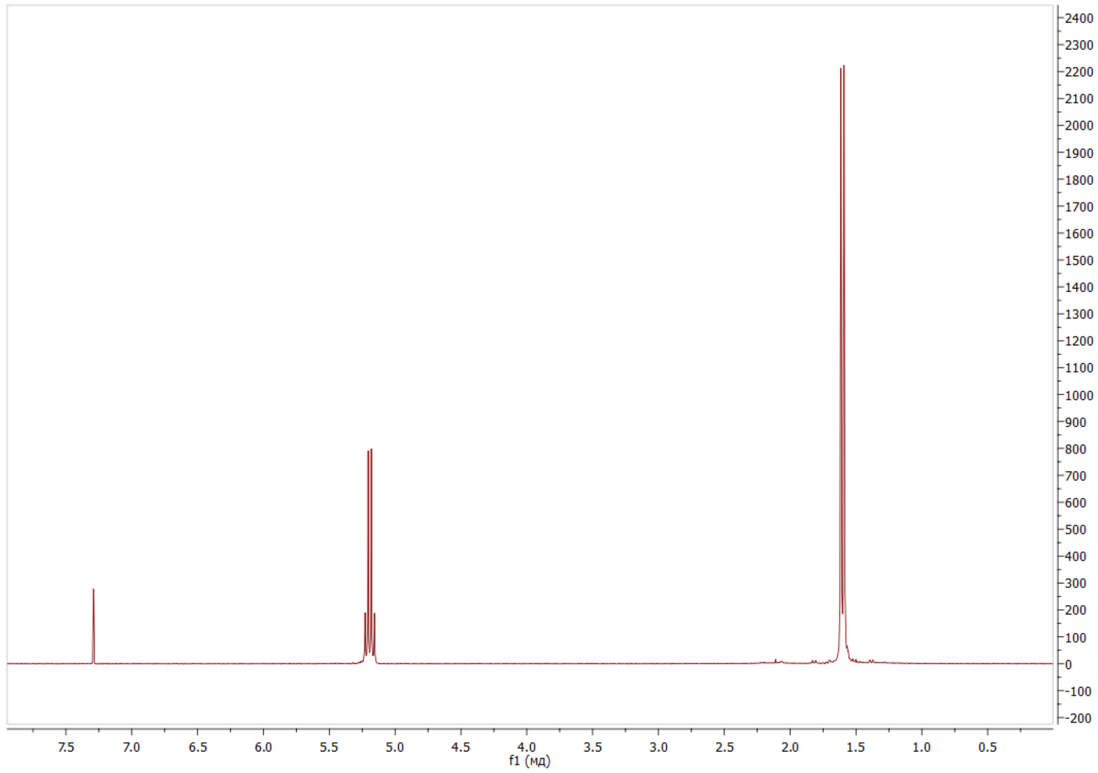

95PLA/5NR, *Sturm method* (d)

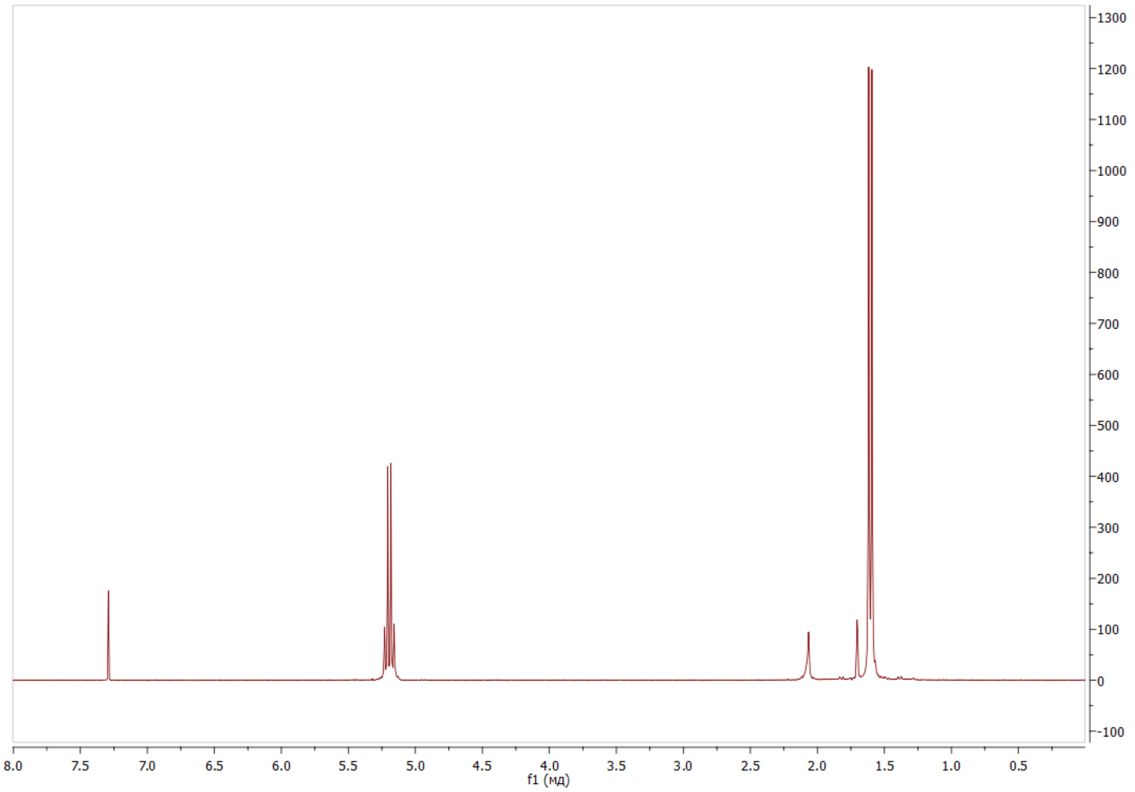

90PLA/10NR, *initial* (e)

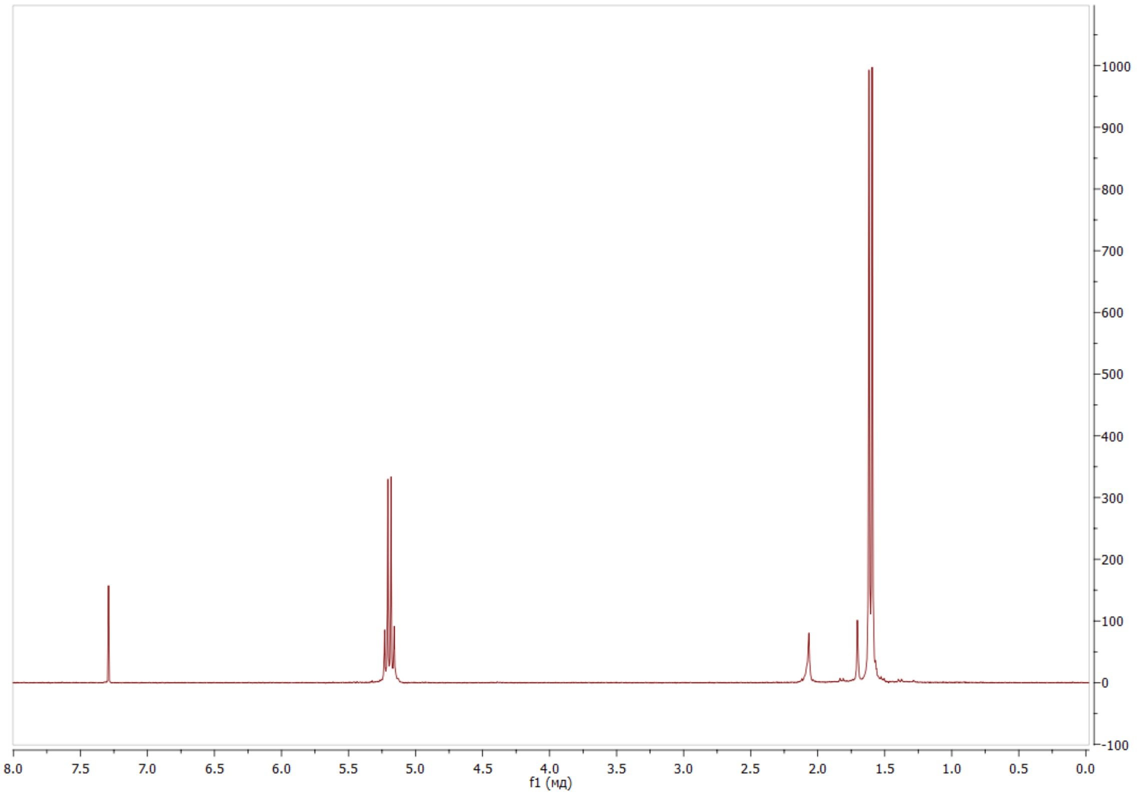

90PLA/10NR, *Sturm method* (f)

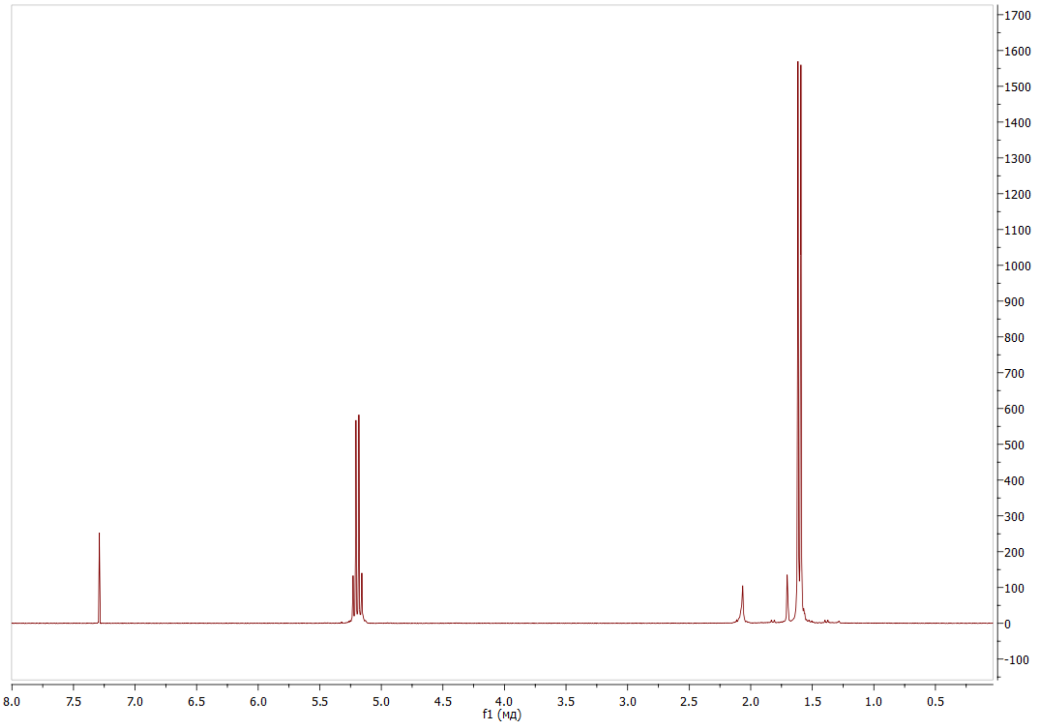

85PLA/15NR, *initial* (g)

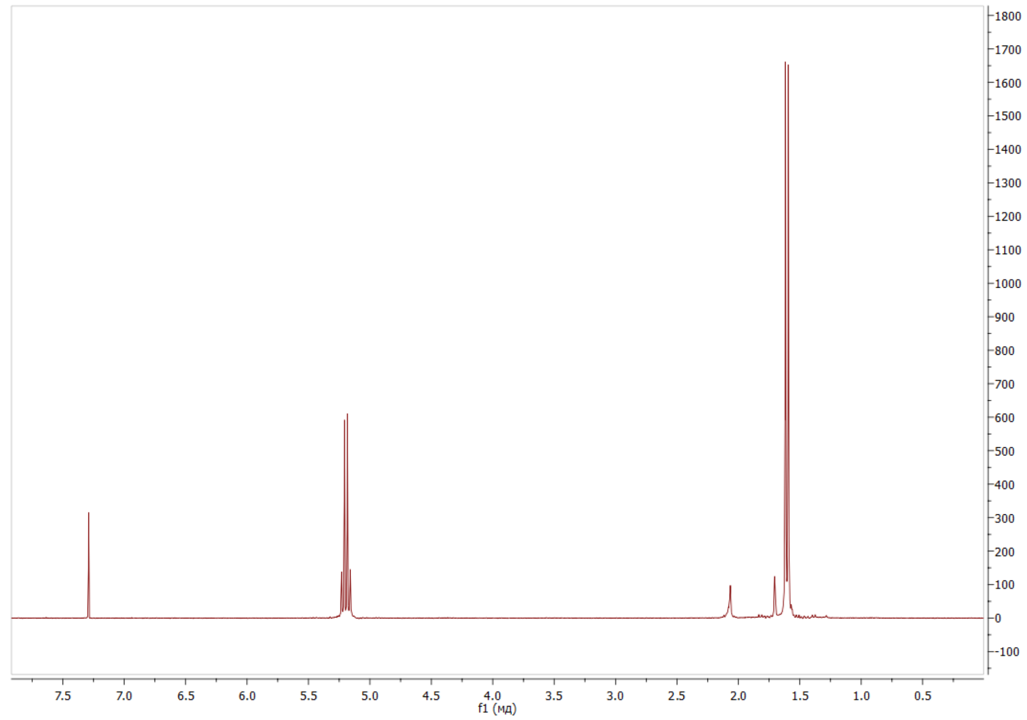

85PLA/15NR, *Sturm method* (h)

Figure S2. ^{1}H NMR spectra of PLA/NR films. The content of NR, wt.%: a,b) 0; c,d) 5; e,f) 10; g,h) 15.

After biotic degradation the NR peaks of 95PLA/5NR sample almost disappeared. In samples with 10 and 15 wt % rubber, the intensity of these peaks somewhat decreases, which confirms the role of NR in the biodegradation of PLA/NR films.
